# Comparison of transcriptional profiles of *Treponema pallidum* during experimental infection of rabbits and *in vitro* culture: Highly similar, yet different

**DOI:** 10.1371/journal.ppat.1009949

**Published:** 2021-09-27

**Authors:** Bridget D. De Lay, Todd A. Cameron, Nicholas R. De Lay, Steven J. Norris, Diane G. Edmondson

**Affiliations:** 1 Department of Pathology and Laboratory Medicine, McGovern Medical School, University of Texas Health Science Center at Houston, Houston, Texas, United States of America; 2 Department of Microbiology and Molecular Genetics, McGovern Medical School, University of Texas Health Science Center at Houston, Houston, Texas, United States of America; Texas A&M University College Station: Texas A&M University, UNITED STATES

## Abstract

*Treponema pallidum* ssp. *pallidum*, the causative agent of syphilis, can now be cultured continuously *in vitro* utilizing a tissue culture system, and the multiplication rates are similar to those obtained in experimental infection of rabbits. In this study, the RNA transcript profiles of the *T*. *pallidum* Nichols during *in vitro* culture and rabbit infection were compared to examine whether gene expression patterns differed in these two environments. To this end, RNA preparations were converted to cDNA and subjected to RNA-seq using high throughput Illumina sequencing; reverse transcriptase quantitative PCR was also performed on selected genes for validation of results. The transcript profiles in the *in vivo* and *in vitro* environments were remarkably similar, exhibiting a high degree of concordance overall. However, transcript levels of 94 genes (9%) out of the 1,063 predicted genes in the *T*. *pallidum* genome were significantly different during rabbit infection versus *in vitro* culture, varying by up to 8-fold in the two environments. Genes that exhibited significantly higher transcript levels during rabbit infection included those encoding multiple ribosomal proteins, several prominent membrane proteins, glycolysis-associated enzymes, replication initiator DnaA, rubredoxin, thioredoxin, two putative regulatory proteins, and proteins associated with solute transport. *In vitro* cultured *T*. *pallidum* had higher transcript levels of DNA repair proteins, cofactor synthesis enzymes, and several hypothetical proteins. The overall concordance of the transcript profiles may indicate that these environments are highly similar in terms of their effects on *T*. *pallidum* physiology and growth, and may also reflect a relatively low level of transcriptional regulation in this reduced genome organism.

## Author summary

The spiral-shaped bacterium that causes syphilis, *Treponema pallidum* subsp. *pallidum*, was first discovered in 1905, but a laboratory system that promotes long-term growth of this tiny organism was not developed until 2017. In this study, we compared the gene expression of *T*. *pallidum* grown in this system to organisms recovered from rabbits infected with the bacterium. Gene expression under these two conditions generally was very similar. However, *T*. *pallidum* grown in rabbits had more RNA ‘messengers’ for genes encoding important cell membrane proteins and protein making machinery, whereas those grown *in vitro* (in glass) had higher RNA levels for genes related to fixing DNA breaks and making vitamins. These gene expression patterns may help us understand how *T*. *pallidum* can cause infections that last for decades and yet can be so hard to grow in the laboratory.

## Introduction

*Treponema pallidum* subsp. *pallidum* (hereafter called *T*. *pallidum*) is the causative agent of syphilis [1–3]. This highly motile spirochete is closely related to the other subspecies of *T*. *pallidum* that cause the non-venereal diseases yaws (subsp. *pertenue*) and bejel (subsp. *endemicum*) [4]. Worldwide, there are an estimated 6 million new cases of syphilis in adults each year, with an additional 300,000 fetal and infant deaths due to congenital syphilis [5,6]. In 2016, the World Health Organization developed a program to decrease the transmission of syphilis by 90% by 2030, with a focus on eliminating congenital syphilis; however, there has recently been a significant increase in new cases of syphilis in North America, Europe, and Asia [6–9]. In the United States alone, there were 35,063 new primary and secondary syphilis cases reported in 2018, representing a 71% increase from 2014 [10]. Coinciding with this increase in primary and secondary syphilis cases, there were 1306 cases of congenital syphilis in 2018 with 78 resulting in still birth and 16 in infant death, reflecting an 185% increase in reported cases from 2014 [7].

The Nichols strain of *T*. *pallidum* was first isolated from a patient from Washington, D.C. with syphilis in 1912 and has been propagated in rabbits since that time [11]. This strain has served as the principal laboratory strain of *T*. *pallidum*; its complete genome was first sequenced in 1998, followed by resequencing in 2013 [12,13]. The genome consists of a single circular chromosome 1.14 Mbp in length with 1,063 predicted protein-encoding open reading frames [13]. Of the predicted open reading frames, only about 55% have predicted functions. Based on sequence information, *T*. *pallidum* lacks the ability to synthesize nucleosides, fatty acids, and most amino acids, as well as proteins necessary for metabolic processes including the Krebs cycle and oxidative phosphorylation [2,12]. Related to its reduced metabolic capabilities, *T*. *pallidum* has numerous genes involved in the transport and utilization of needed molecules from the host [1,14,15].

The genomic sequences of many additional strains of *T*. *pallidum* subsp. *pallidum*, subsp. *pertenue*, subsp. *endemicum*, and *Treponema paraluiscuniculi* (venereal spirochetosis of rabbits) reveal that these organisms are all closely related, with ~99.2% sequence identity among the *T*. *pallidum* subspecies and 98.1% identity between *T*. *pallidum* and *T*. *paraluiscuniculi* strains (reviewed in [16]). Moreover, the gene content in this group of organisms is virtually identical, with heterogeneities consisting primarily of single nucleotide polymorphisms and duplications of *T*. *pallidum* repeat (*tpr*) genes. Within *T*. *pallidum* subsp. *pallidum*, the strains are subdivided into at least two genetic clusters, consisting of isolates more closely related to the Nichols strain and others related to the SS14 strain [17–19]. This observation suggests a relatively recent divergence among syphilis-causing organisms.

Although the Nichols strain of *T*. *pallidum* has been propagated in rabbits and other animals for over a century, it has only recently been successfully cultured *in vitro* [20,21]. Co-culture of *T*. *pallidum* strains *in vitro* with Sf1Ep cottontail rabbit epithelial cells in growth media based on Eagle’s Minimal Essential Medium (MEM) under microaerobic conditions (1.5% O_2_, 5% CO_2_) at 34°C resulted in up to 100-fold increase of *T*. *pallidum*, but serial passage of *T*. *pallidum* remained unsuccessful and cultures generally survived for less than 18 days [14]. In 2018, the first successful long-term *in vitro* cultivation of *T*. *pallidum* was reported [22]. This culture system uses a modified culture medium (TpCM-2, containing CMRL 1066 medium as its basal medium) in combination with Sf1Ep cells grown under microaerobic conditions to successfully cultivate *T*. *pallidum* continuously *in vitro*, with retention of infectivity in the rabbit model [22–25].

The recent advancement in the ability to cultivate *T*. *pallidum* in long-term *in vitro* culture has opened up the possibility of studying the biology of this enigmatic organism in greater detail. Although gene expression has been examined previously to some extent in rabbit-propagated *T*. *pallidum* [2,26,27], it is uncertain how gene expression is affected by *in vitro* culture. This study compares the gene transcript levels of *T*. *pallidum* propagated by intratesticular infection of rabbits with those of *T*. *pallidum* grown *in vitro* utilizing global RNA-seq analysis and quantitative reverse transcriptase PCR (qRT-PCR) of a small subset of genes. Overall, expression of 91% of the *T*. *pallidum* genes was not significantly different between these two culture conditions. These results indicate that *in vitro* cultivation of *T*. *pallidum* is a useful alternative to rabbit infection for studying gene expression patterns and other biological properties of this important human pathogen. In addition, the data support the concept that *T*. *pallidum* may have a limited capability to alter gene expression in response to varying environmental conditions.

## Results

### Global transcriptional profiles of *T*. *pallidum* propagated in rabbits and in *in vitro* cultures

**Fig 1:**
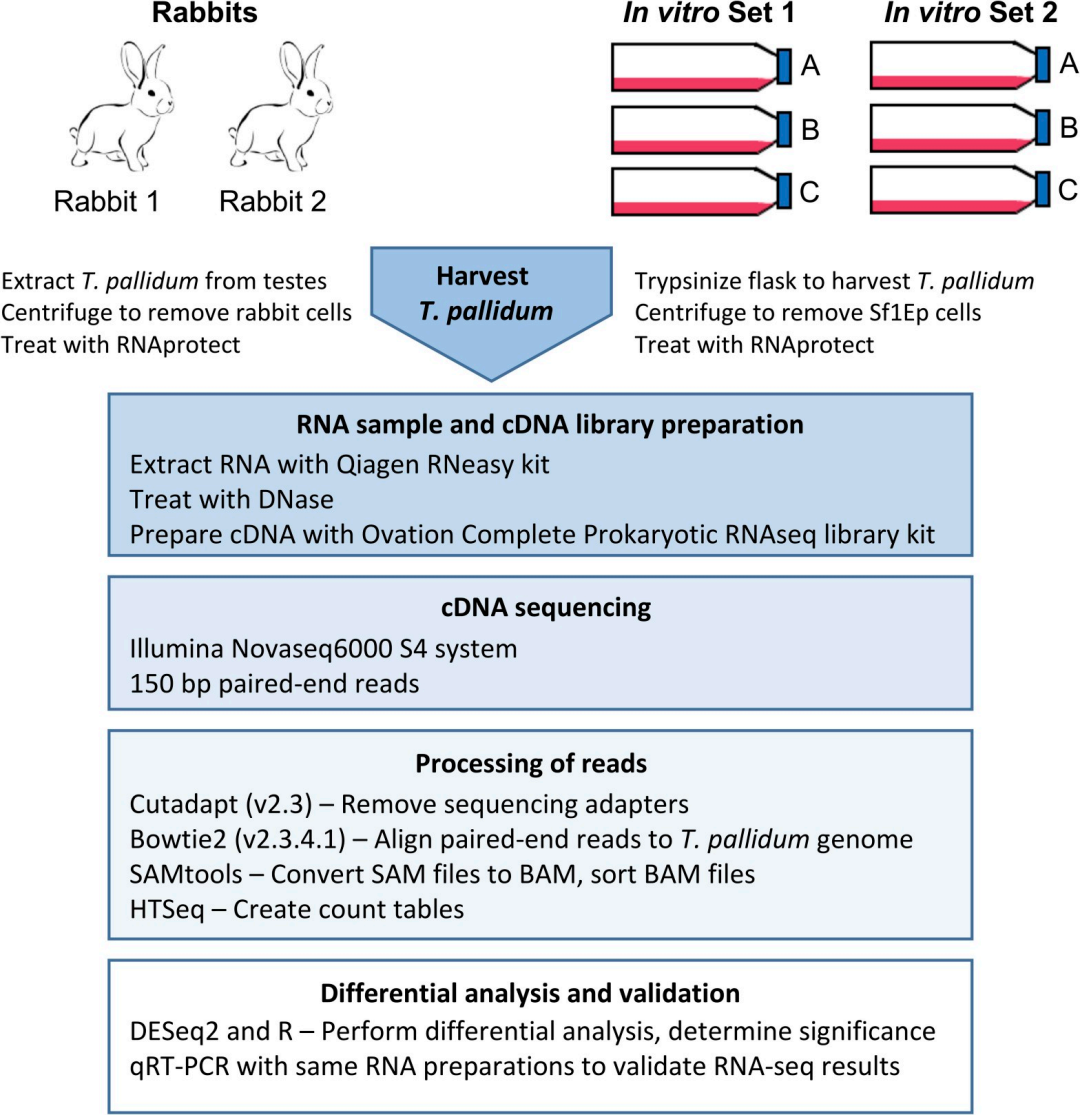
Schematic diagram of the methods used in this study.

All of the experiments in this study utilized the Nichols strain of *T*. *pallidum* subsp. *pallidum*, hereafter referred to as *T*. *pallidum*. Two sets of *in vitro* samples, each consisting of *T*. *pallidum* RNA collected from three 75-cm^2^ flask cultures, were compared to *T*. *pallidum* RNA collected from two rabbits (Fig 1). RNA sequences obtained using an RNA-seq approach were mapped against the published *T*. *pallidum* genome (NC_021490). An average of 46,823,419 read pairs were obtained from each of the six *in vitro* samples, in comparison to an average of 44,265,095 read pairs from each rabbit sample (Table 1). The overall percentage of read pairs that mapped to the *T*. *pallidum* genome differed between *in vitro* and rabbit samples with an average of 12.5% of the *in vitro* read pairs mapping to the *T*. *pallidum* genome in comparison to 71.4% of the rabbit sample read pairs (Table 1). Both the *in vitro* and rabbit samples contained residual mammalian cells prior to RNA extraction.

**Table 1:** Read pairs generated from the *in vitro* culture and rabbit-propagated samples by RNA-seq analysis. The total number of read pairs per sample and the number of read pairs that map to the *T*. *pallidum* genome (NC_021490) were generated by HTSeq.

Sample	Total read pairs	Read pairs mapping to *T*. *pallidum* genome	Percentage of read pairs mapping to *T*. *pallidum* genome
***In vitro* set 1A**	69,512,164	9,616,921	13.8
***In vitro* set 1B**	46,264,513	5,773,537	12.5
***In vitro* set 1C**	42,629,508	5,356,627	12.6
***In vitro* set 2A**	50,603,212	5,085,803	10.1
***In vitro* set 2B**	37,361,899	4,759,202	12.7
***In vitro* set 2C**	34,569,218	4,397,865	12.7
**Rabbit set 1**	52,167,004	41,316,442	79.2
**Rabbit set 2**	36,371,185	21,935,020	60.3

Read pairs that mapped to the *T*. *pallidum* genome were assigned to individual accession numbers by HTSeq with the minimum alignment quality set to 0 (Tables 2 and S1). The majority of the assigned read pairs corresponded to rRNA sequences, with an average of 84.0% of the assigned *in vitro* culture read pairs and 91.4% of the rabbit infection-derived read pairs corresponding to rRNA. The average percent read pairs per *in vitro* sample that mapped to protein-encoding genes (16.0%) was almost twice as high as the average percent per rabbit sample (8.6%). Similarly, the average percent of tRNA read pairs per sample in the *in vitro* samples (0.08%) was also higher than in the rabbit samples (0.02%).

**Table 2:** Average number of read pairs from long-term *in vitro* culture and rabbit sample sets that map to the *T*. *pallidum* genome. The *in vitro* sample set consists of the average number of read pairs from six individual RNA samples. The rabbit set consists of the average number of read pairs from RNA samples collected from two different rabbits.

Sample set	Average assigned read pairs per sample	Average unassigned* read pairs per sample	Average rRNA read pairs per sample	Average mRNA read pairs per sample	Average tRNA read pairs per sample
*In vitro*	5,831,659 (12.5%)	40,991,760 (87.5%)	4,895,988 (84.0%)	930,826 (16.0%)	4,846 (0.08%)
Rabbit	31,625,731 (71.4%)	12,643,364 (28.6%)	28,900,229 (91.4%)	2,717,615 (8.6%)	7,887 (0.02%)

* The *in vitro* culture system utilizes Sf1Ep cottontail rabbit (*Sylvilagus floridanus*) epithelial cells, which were present in the sample after trypsinization of the culture to harvest *T*. *pallidum*. Likewise, the RNA sample obtained from New Zealand white rabbits (*Oryctolagus cuniculus*) contained residual rabbit tissue cells after *T*. *pallidum* was extracted from the rabbit testes. Centrifugation reduces, but does not eliminate, the presence of the eukaryotic cells in these preparations. Therefore, the read pairs that did not map to the *T*. *pallidum* genome likely represent Sf1Ep cottontail rabbit cell RNA sequences and New Zealand white rabbit RNA sequences in the *in vitro* and rabbit- culture-propagated groups, respectively.

### Consistency of RNA-seq read profiles

**Fig 2:**
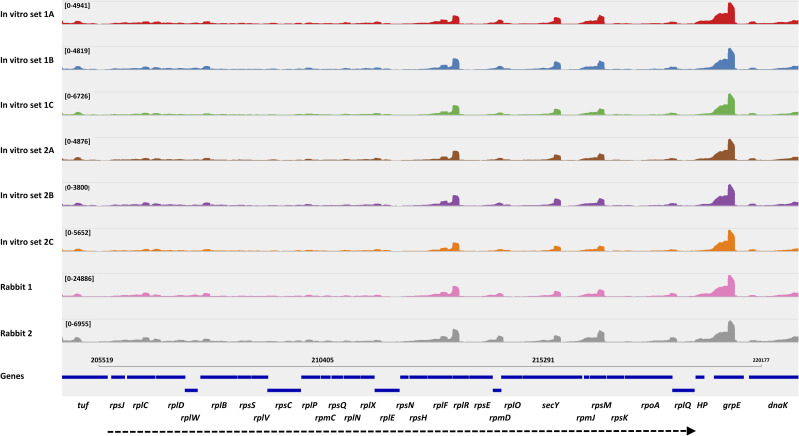
**High consistency of RNA-seq read coverage patterns among 8 independently processed RNA samples, as exemplified by the large ribosomal protein gene operon region of *T*. *pallidum* (dashed arrow at bottom).** Coverage patterns are shown for the 6 *in vitro* culture specimens (in vitro sets 1A-1C and 2A-2C) and the specimens from infected rabbits 1 and 2. The read coverage graphic was prepared using the Sashimi plot feature of the Integrative Genomic Viewer (IGV) program.

RNA-seq is considered a valuable measure of global RNA transcript levels. However, RNA-seq read coverage profiles exhibit a surprising degree of unevenness of transcript levels within genes and operons. To examine whether differences in read frequencies between *T*. *pallidum* propagated by *in vitro* culture or rabbit infection could be due in part to differential RNA stability, we scanned the coverage profile throughout the genome using the Integrative Genomics Viewer (IGV) [28] and its appended Sashimi program [29]. The coverage profiles of the eight RNA preparations from *in vitro*- and rabbit-propagated *T*. *pallidum* were found to be remarkably similar; this observation is exemplified by the near identical patterns found in the vicinity of the large ribosomal protein gene operon (Fig 2). To quantitatively compare coverage profiles between samples, the read distributions within the length of all the protein-encoding genes were determined and averaged for each RNA preparation. Highly similar read distribution profiles were obtained from RNA samples obtained from infected rabbits or *in vitro* cultures. Coverage profiles were highest at the 5’ regions and lowest, at about 80–85% of maximum, in the 3’ regions. Overall, the similar read distribution profiles in all samples indicate that it is unlikely that differences in read distribution significantly contributed to gene expression differences. Thus the observed differences in normalized average counts are unlikely to be the result of differences in RNA degradation patterns (e.g. altered expression or activity of RNases) or related effects.

### Comparison of the most highly expressed *T*. *pallidum* genes during rabbit infection and *in vitro* growth

**Fig 3:**
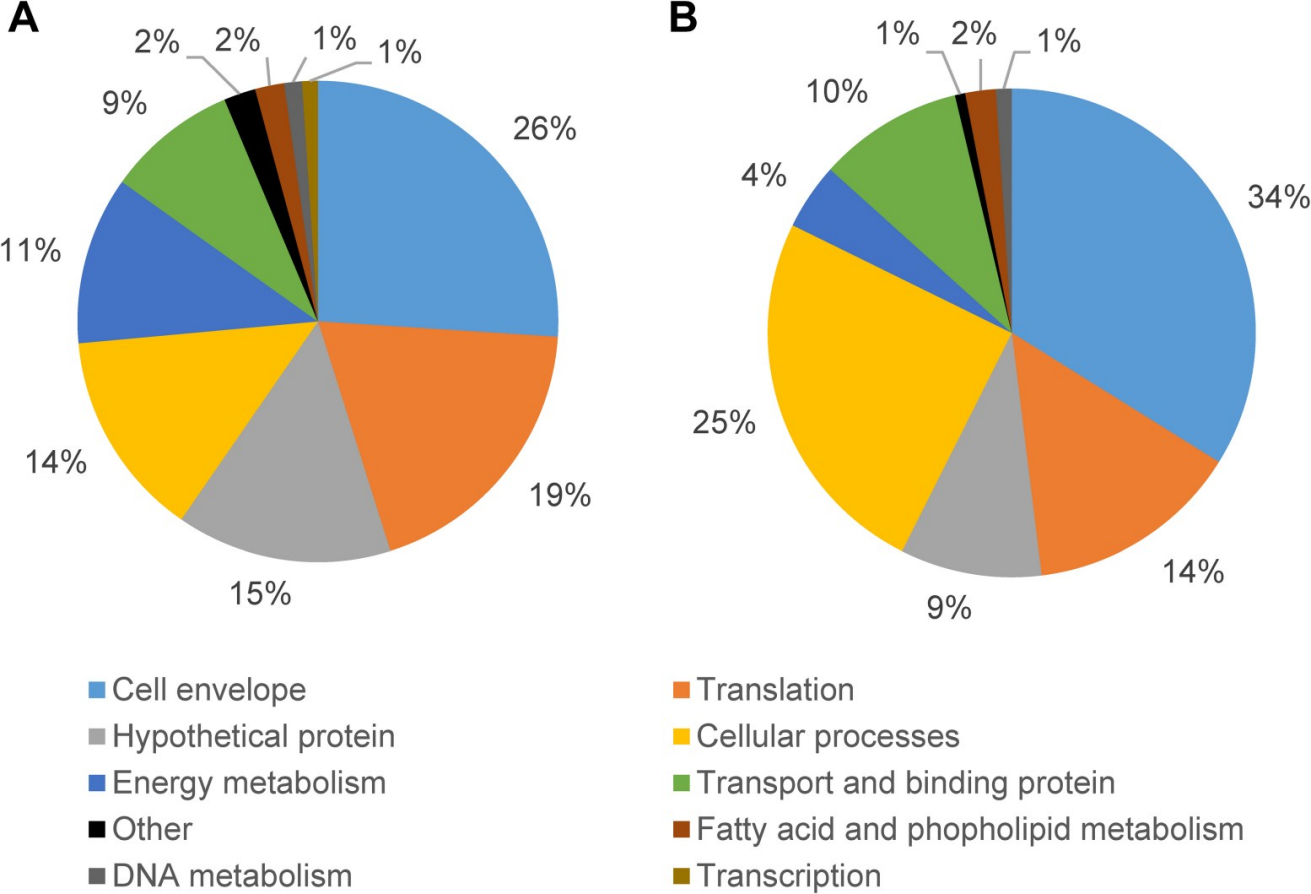
Functional roles of the fifty *T*. *pallidum* genes most highly expressed in rabbits and *in vitro*. (A) Functional roles of the fifty *T*. *pallidum* genes with the highest expression during rabbit infection. (B) Functional roles of the fifty *T*. *pallidum* genes most highly expressed during *in vitro* culture.

The fifty *T*. *pallidum* protein-encoding genes with the highest transcript levels during rabbit infection and long-term *in vitro* culture (Table 3) were determined by calculating FPKM (fragments per thousand bases per million reads) for each gene using the counts generated by HTSeq. Genes encoding RNA products (rRNAs and tRNAs) were excluded from this analysis. The functional group corresponding to each of these genes was assigned based on the predicted functions of *T*. *pallidum* genes [12], and the percentage of each functional group for the top fifty most highly expressed genes was determined (Fig 3). The overall functional group percentages for both rabbit infection and *in vitro* culture derived *T*. *pallidum* were highly similar. The highest frequency functional group for both rabbit (Fig 3A) and *in vitro* culture (Fig 3B) was cell envelope proteins, comprising 26% and 34% of the top 50 most highly expressed genes in rabbit and *in vitro* cultivation, respectively; this group includes flagellar proteins, membrane lipoproteins, and other membrane-associated proteins. Other functional groups with high transcript levels (>2% of total) included those encoding proteins involved in translation, cellular processes (including chaperones and proteins involved in oxidative/reduction reactions), energy metabolism, transport and substrate binding, and unknown functions (hypothetical proteins) (Fig 3). Among these groups, the categories exhibiting the highest and lowest ratio of total transcripts (rabbit infection/*in vitro* culture) were energy metabolism (2.8) and cellular processes (0.6).

**Table 3:** *T*. *pallidum* protein-encoding genes with the highest average FPKM during *in vitro* culture or rabbit infection. The fifty genes with the highest gene expression based on average FPKM during *in vitro* culture and rabbit infection are listed in order from highest expression to lowest in the rabbit-derived specimens. ORF numbers in black indicate that the gene is one of the fifty most highly-expressed genes during both rabbit infection and *in vitro* culture. ORFnumbersinblue indicate that the gene is one of the fifty most highly-expressed genes in rabbits but not *in vitro* culture, while ORFnumbersinorange indicate indicate that the gene is one of the fifty most highly-expressed during *in vitro* culture, but not in rabbit infection. Average FPKM was calculated based on counts determined by HTSeq with the minimum alignment quality set to 0 and excluding rRNA and tRNA read pairs. Unless otherwise indicated, functional categories based on [26].

*T*. *pallidum* ORF number	Gene ID	Functional category^a^	Average FPKM *in vitro*	Average FPKM in rabbit
TPANIC_0792	*flaB2*	Cell envelope; Surface structures	4,830	2,223
TPANIC_0509	*ahpC*	Cellular processes; detoxification	8,013	2,187
TPANIC_RS05235	*ssrA*	Transport and binding proteins; Amino acids, peptides, amines*	4,698	1,954
TPANIC_0974	*flgM*	Cell envelope; Surface structures*	2,703	1,834
TPANIC_1013	*groES*	Cellular processes; Chaperones	2,530	1,338
TPANIC_RS05155	*rnpB*	Translation; tRNA modification*	2,159	1,293
TPANIC_0061	*rpsR*	Translation; Ribosomal protein synthesis and modification	1,762	1,266
TPANIC_0844	*gap*	Energy metabolism; Glycolysis/gluconeogenesis	1,965	1,223
TPANIC_0525	*efp*	Translation; Translation factors	2,439	1,138
TPANIC_0684	*mglB-2*	Transport and binding proteins; Carbohydrates, organic alcohols, acids	1,434	1,130
TPANIC_0870	*flaB3*	Cell envelope; Surface structures	1,213	1,088
TPANIC_0768	*tmpA*	Cell envelope; Other	1,240	870
TPANIC_RS01150	*bioY*	Cell envelope; Biotin transmembrane transporter activity*	1,245	783
TPANIC_0919	*trx*	Energy metabolism; Electron transport	690	659
TPANIC_0215	*grpE*	Cellular processes; Chaperones	1,258	645
TPANIC_0277	*ctp*	Translation; Degradation of proteins, peptides, glycopeptides	925	635
TPANIC_0777	hypothetical protein	Hypothetical protein	906	598
TPANIC_0746	*ppdk*	Energy metabolism; Glycolysis/gluconeogenesis	683	557
TPANIC_RS00240	M23 family metallopeptidase	Translation; Degradation of proteins, peptides, glycopeptides*	684	549
TPANIC_0868	*flaB1*	Cell envelope; Surface structures	1,207	545
TPANIC_0858	UPF0164 family protein	Hypothetical protein	730	540
TPANIC_0398	*fliE*	Cell envelope; Surface structures	925	537
TPANIC_0486	antigen, p83/100	Cell envelope; Biosynthesis of surface polysaccharides, lipopolysaccharides	1,346	502
TPANIC_0973	*pheS*	Translation; Amino acyl tRNA synthetases	685	499
TPANIC_RS04705	TraB/GumN family protein	Hypothetical protein	613	491
TPANIC_1029	DbpA RNA binding domain protein	Translation: rRNA processing*	548	487
TPANIC_0750	von Willebrand factor type A domain protein	Hypothetical protein*	1,088	485
TPANIC_0770	DEAD/DEAH box helicase	Translation; Translation factors	779	480
TPANIC_1032	NusG domain II-containing protein	Hypothetical protein*	453	480
TPANIC_0352	YdbC family protein	Hypothetical protein*	1,277	467
TPANIC_0153	divergent PAP2 family protein	Hypothetical protein*	614	459
TPANIC_0303	*mutL*	DNA metabolism; Replication, recombination, repair	1,108	419
TPANIC_0272	*soj*	Other; Adaptations and atypical conditions	726	414
TPANIC_0122	*pckA*	Energy metabolism; Glycolysis/gluconeogenesis	520	409
TPANIC_0249	*flaA-1*	Cell envelope; Surface structures	606	405
TPANIC_0824	*tktB*	Energy metabolism; Pentose phosphate pathway	768	402
TPANIC_0505	*hxk*	Energy metabolism; Glycolysis/gluconeogenesis	289	376
TPANIC_RS01765	hypothetical protein	Hypothetical protein*	366	369
TPANIC_0709	*whiG*	Transcription; DNA-dependent RNA polymerase	797	369
TPANIC_0098	*dnaJ1*	Cellular processes; Chaperones	681	364
TPANIC_RS01810	hypothetical protein	Hypothetical protein*	804	351
TPANIC_0462	hypothetical protein	Hypothetical protein	495	351
TPANIC_0361	1-acyl-sn-glycerol-3-phosphate acyltransferase	Fatty acid and phospholipid metabolism; Other	704	350
TPANIC_0807	*rpmF*	Translation; Ribosomal proteins: synthesis and modification	410	345
TPANIC_0547	*lytB*	Cellular processes; Toxin production and resistance	827	343
TPANIC_0257	*glpQ*	Fatty acid and phospholipid metabolism; Degradation	727	336
TPANIC_0356	RNA-binding protein	Other; Unknown	517	336
TPANIC_0925	flavodoxin	Energy metabolism; Electron transport	468	332
TPANIC_0663	*flaA2*	Cell envelope; Other	445	329
TPANIC_0956	TRAP transporter TatT component family protein	Transport and binding proteins; lipids	445	327
TPANIC_0435	*tpp17*	Cell envelope; Lipoproteins	1,059	306
TPANIC_0432	hypothetical protein	Hypothetical protein*	875	291
TPANIC_0362	*rpmB*	Translation; Ribosomal proteins: synthesis and modification	881	258
TPANIC_0737	*msmE*	Transport and binding proteins; Carbohydrates, organic alcohols, acids	723	291
TPANIC_0751	*vwb*	Cell envelope; Surface structures*	643	224
TPANIC_0928	hypothetical protein	Hypothetical protein	647	211
TPANIC_0652	*potA*	Transport binding proteins; Amino acids, peptides, amines	620	277
TPANIC_0731	*nudE*	Cellular processes; Signal transduction*	595	307
TPANIC_0358	glycoside hydrolase family 57 protein	Energy metabolism; Glycolysis/gluconeogenesis*	595	260
TPANIC_0748	*cfpA*	Cell envelope; Surface structures	596	322
TPANIC_0417	*cutE*	Cellular processes; Protein and peptide secretion	576	136

* Functional roles based on Gene Ontology (GO) terms (QuickGO).

### Comparison to previous *T*. *pallidum* RNA and protein expression data

The 50 most highly expressed genes from this experiment were then compared to the 50 most highly expressed *T*. *pallidum* genes during rabbit infection previously reported by Šmajs et al. [26] in a microarray study. After accounting for newly annotated genes, 42% (18/43) and 30% (13/44) of the most highly expressed genes during rabbit infection and *in vitro* culture in this study were also among the most highly expressed in the previous microarray transcriptome study (S2 Table) [26]. The most common functional groups for the top 50 most highly expressed *T*. *pallidum* genes in rabbits as determined by Šmajs et al. [26] were hypothetical proteins (34%), cell envelope (26%), translation (14%), energy metabolism (12%), and cellular processes (8%). These results were similar to the data obtained for this study, with the most common functional groups for both the rabbit infection and *in vitro* cultivation samples being cell envelope, translation, cellular processes, and hypothetical proteins (Fig 3).

Like in the previous work, we found that the four genes encoding flagellar filament proteins (*flaB1-3*, *flaA*) were among the most highly expressed genes in rabbit infection and *in vitro* cultivation, but unlike in the previous work the cytoplasmic filament protein *cfpA* was not one of the most highly expressed genes in our study (Table 3). Of the genes encoding outer membrane proteins or lipoproteins, two (TPANIC_0663 and *tmpA*) were among the most highly expressed genes in our results as well as in the previous work, while five additional membrane components were detected in the top 50 most highly expressed genes in the previous rabbit transcriptome data (*tp34*, *tmpC*, *tpp15*, *tmpB*, and *tpp17*). Although three genes encoding chaperonins were among the most highly expressed in the previous work (*groEL*, *groES*, and *dnaK*), they were not among the most highly expressed genes in this study. Genes responsible for the maintenance of redox potential (*ahpC*, flavodoxin, thioredoxin), a V-type ATPase component (TPANIC_0424), and glyceraldehyde-3-phosphate dehydrogenase (TPANIC_0844) were all highly expressed in this study as well as in the previous transcriptome study.

To further compare the data generated by RNA-seq to the previously reported microarray data, scatter plots were generated based on the reported cDNA/DNA ratio values from the microarray work and the FPKM values generated in this study. Overall, there was a low concordance between the prior microarray data and the data generated by RNA-seq; the microarray data was somewhat more similar to the rabbit infection RNA-seq results (R^2^ = 0.28) than to the *in vitro* culture results (R^2^ = 0.19).

Osbak et al. [30] analyzed the proteome of *T*. *pallidum* subsp. *pallidum* DAL-1 in a semi-quantitative manner using mass spectroscopy. A total of 557 proteins (corresponding to 54% of the predicted protein-encoding genes) were identified by this means. In their study, the abundance of these proteins as measured by the normalized spectral abundance factor (NSAF) did not correlate with transcript abundance as determined previously in the Šmajs et al. [26] microarray analysis. Similarly, we found that the protein NSAF values did not correlate well with the RNA transcript levels determined for *T*. *pallidum* propagated in infected rabbits or *in vitro* cultures, yielding R^2^ values of 0.006 and 0.0028, respectively.

### Differential gene expression of *T*. *pallidum* cultured in rabbits and *in vitro*

**Fig 4:**
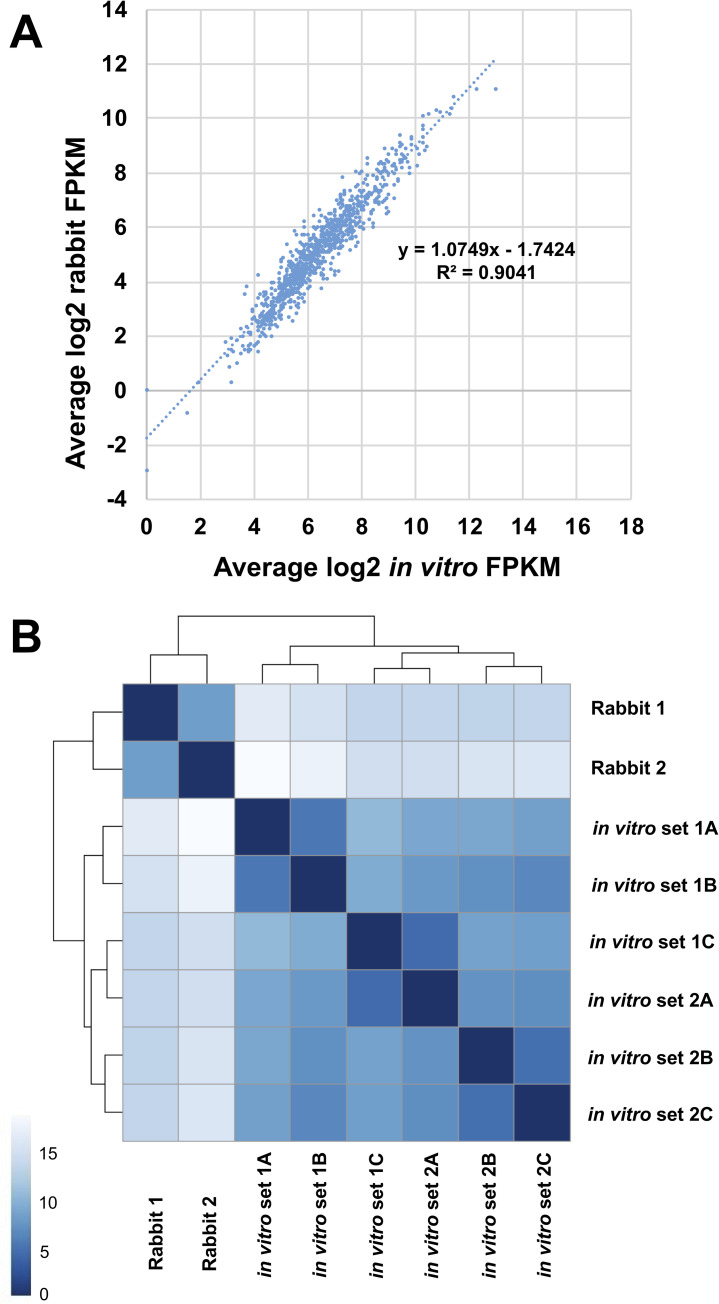
Similarity of *T*. *pallidum* transcript levels between samples collected from infected rabbits and from *in vitro* culture. A) Scatter plot comparing average log2-transformed FPKM values of *T*. *pallidum* collected during rabbit infection and *in vitro* culture. B) Poisson distance matrix based on pairwise DESeq2 differential expression analysis showing that RNA samples taken from the two individual rabbits group together, while the six *in vitro* culture samples group separately. Darker blue squares indicate that samples are more closely related than those with lighter blue squares.

A scatter plot comparing the log_2_-transformed average FPKM values for rabbit infection and *in vitro* culture was created to compare the similarity between these two growth conditions (Fig 4A). There was a high concordance between the RNA-seq data generated for rabbit infection and *in vitro* culture (R^2^ = 0.90), indicating that there is not a large difference in *T*. *pallidum* gene expression between these two culture conditions. A Poisson distance matrix was calculated from rlog-transformed read counts to compare gene expression of the two rabbit samples to the six *in vitro* samples. The gene expression profiles of the two rabbit samples were most similar to each other, and the six *in vitro* samples also clustered together (Fig 4B).

**Fig 5:**
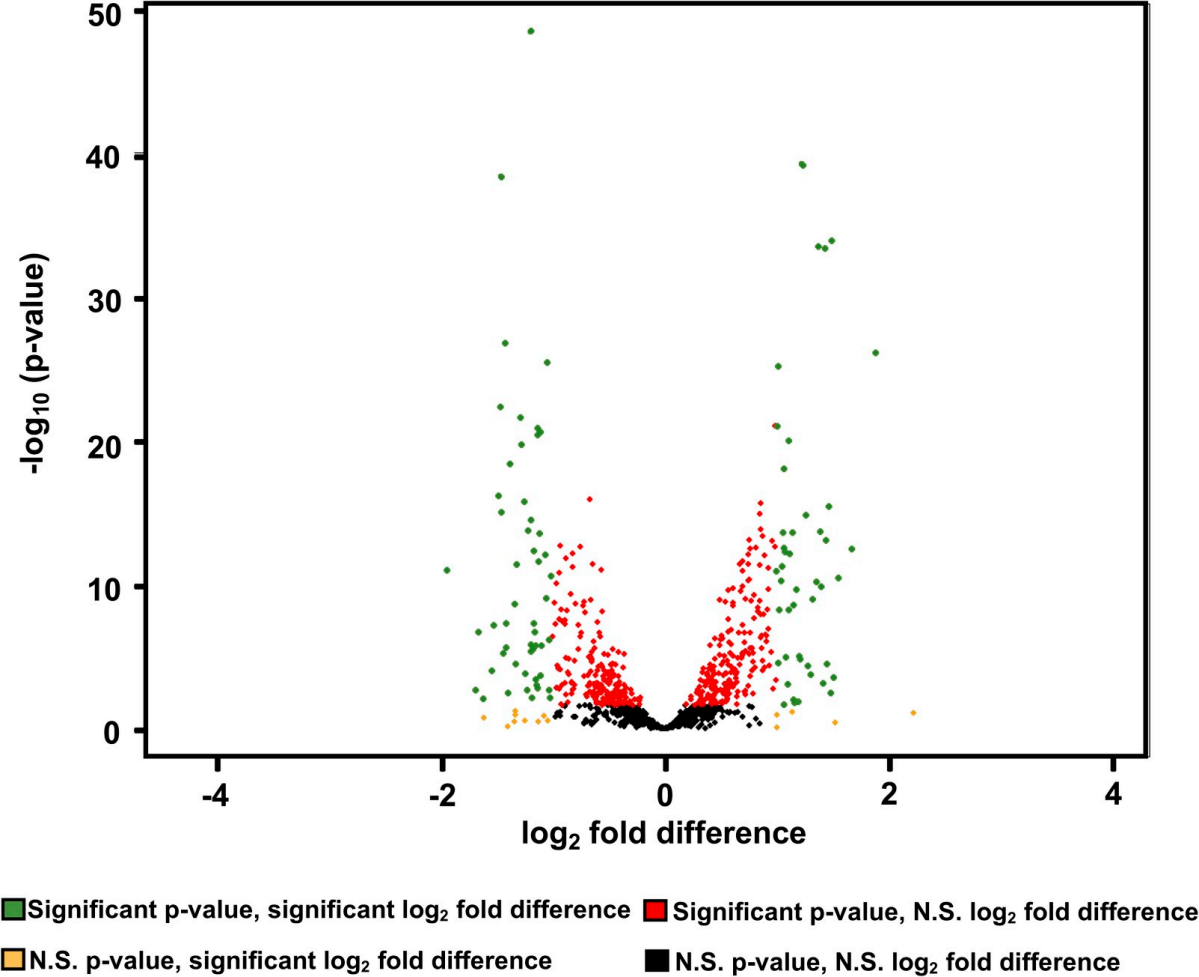
Identification of genes with significantly different transcript levels between *T*. *pallidum* during *in vitro* culture vs. rabbit infection. The volcano plot indicates significantly differentially expressed genes between *T*. *pallidum* grown *in vitro* and in rabbits, as determined by DESeq2. Log_2_-fold difference values reflect the ratio of rabbit/culture transcript levels. Significantly differentially-expressed genes, with false discovery rate (FDR) adjusted p-value ≤ 0.05 and |log_2_-fold difference| of ≥ 1 are indicated in green. N.S. indicates the p value is not significant (>0.05).

Differential expression analysis was then used to compare the individual gene transcript levels from the combined rabbit samples to the combined *in vitro* samples (S2 Table), omitting tRNA transcripts. Genes were considered to be significantly differentially expressed if the |log_2-_fold difference| was ≥ 1 (equivalent to a 2-fold difference in gene expression) and the false discovery rate (FDR) adjusted p-values were ≤ 0.05. Of the 1063 genes from the *T*. *pallidum* genome that were represented by the RNA sequencing data, 94 (9%) were differentially expressed (Fig 5 and Table 4). To verify these results, a subset of significantly differentially expressed genes were subjected to qRT-PCR. All of the genes examined by qRT-PCR were differentially expressed (p ≤ 0.05) between *T*. *pallidum* grown *in vitro* and in rabbits, in agreement with the RNA-seq results (Table 5). The RNA-seq and qRT-PCR differential expression values in Table 5 exhibited a high degree of correlation (r = 0.95).

**Table 4:** Genes with significantly different transcript levels in *T*. *pallidum* from *in vitro* cultures and infected rabbits. Average normalized counts, log_2_-fold difference and false discovery rate (FDR) adjusted p-values were determined using DESeq2. Gene function was based on [9]. Functional categories were based on Gene Ontology (GO) terms (QuickGO). Log_2_ values represent the ratio of rabbit counts/*in vitro* counts. Genes are listed in the order of ascending log_2_-fold Difference. Differences in tRNA expression were excluded from this analysis.

*T*. *pallidum* ORF number	Gene ID	Functional category	Average normalized count *in vitro*	Average normalized count in rabbit	Log_2_ fold difference (rabbit/ in vitro)	Adjusted p-value
TPANIC_RS05180	hypothetical protein	Hypothetical protein	165	43	-1.76	8.09E-09
TPANIC_RS05190	hypothetical protein	Cellular component; Cell membrane*	38	12	-1.75	.001
TPANIC_RS05315	hypothetical protein	Hypothetical protein	45	16	-1.59	7.68E-05
TPANIC_0340	*folC*	Biosynthesis of cofactors, prosthetic groups, carriers; Biotin	415	144	-1.53	1.08E-19
TPANIC_0064	hypothetical protein	Hypothetical protein	367	123	-1.51	1.98E-20
TPANIC_0693	hypothetical protein	Hypothetical protein	1,153	415	-1.48	1.24E-30
TPANIC_0258	hypothetical protein	Hypothetical protein	632	246	-1.47	3.19E-15
TPANIC_0636	*recO*	DNA metabolism; DNA replication, recombination, repair	87	32	-1.46	8.75E-06
TPANIC_RS04015	acyl-CoA ligase	Fatty acid and phospholipid metabolism*	1,448	539	-1.44	6.37E-26
TPANIC_0140	*ntpJ*	Transport and binding proteins; Cations	538	207	-1.42	8.49E-18
TPANIC_0516	*mviN*	Other categories; Adaptations and atypical conditions	92	34	-1.42	.004
TPANIC_0327	*skp*	Cell envelope; Other	433	179	-1.37	2.09E-10
TPANIC_RS00640	hypothetical protein	Hypothetical protein	81	29	-1.36	3.77E-06
TPANIC_1010	*ndk*	Purines, pyrimidines, nucleosides, nucleotides; Nucleoside and nucleotide interconversion	313	119	-1.36	5.57E-12
TPANIC_0637	*miaA*	Translation; tRNA modification	149	61	-1.31	4.81E-08
TPANIC_0405	penta-peptide repeat protein	Cellular processes; Toxin production and resistance	361	147	-1.30	6.04E-17
TPANIC_0592	M15 family metallopeptidase	Peptidase*	603	240	-1.28	1.13E-19
TPANIC_0355	hypothetical protein	Hypothetical protein	99	40	-1.27	6.75E-05
TPANIC_0670	*ddl*	Cell envelope; Biosynthesis of murein sacculus and peptidoglycan	382	157	-1.26	3.15E-07
TPANIC_RS04020	hypothetical protein	Hypothetical protein	174	72	-1.24	5.61E-07
TPANIC_0283	*coaD*	Cell envelope; Biosynthesis of polysacch and lipopolysacch	93	37	-1.23	.0004
TPANIC_RS00645	hypothetical protein	Cellular component; Cell membrane*	58	26	-1.20	1.22E-05
TPANIC_0162	*ruvB*	DNA metabolism; DNA replication, recombination and repair	2,494	1,108	-1.18	1.05E-14
TPANIC_0328	*mutS*	DNA metabolism; DNA replication, recombination and repair	3,966	1,746	-1.18	5.10E-41
TPANIC_RS05385	hypothetical protein	Hypothetical protein	47	21	-1.18	.008
TPANIC_0130	hypothetical protein	Cell envelope; Surface structures	1,408	607	-1.18	2.70E-13
TPANIC_0027	predicted CorC Co/Mg efflux protein [35]	Cellular processes; Toxin production and resistance	191	84	-1.17	.0007
TPANIC_0841	*htrA2*	Translation; Degradation of proteins, peptides and glycopeptides	1,010	447	-1.16	7.09E-25
TPANIC_0902	*est*	Fatty acid and phospholipid metabolism; Degradation	680	320	-1.16	1.17E-14
TPANIC_RS05290	50S ribosomal protein L28	Ribosomal proteins; Synthesis and modification	90	38	-1.16	3.73E-06
TPANIC_0491	*mltG*	Cell envelope; Biosynthesis of murein sacculus and peptidoglycan*	364	155	-1.16	1.67E-15
TPANIC_0156	acyl-CoA thioesterase	Fatty acid metabolism; Acyl-CoA hydrolase activity*	139	59	-1.16	6.01E-07
TPANIC_0456	hypothetical protein	Hypothetical protein	4,754	2,014	-1.14	3.02E-14
TPANIC_0840	MFS transporter	Cell envelope; transmembrane transport*	821	381	-1.12	5.67E-20
TPANIC_0065	*rsmD*	Translation*	98	47	-1.09	1.07E-05
TPANIC_0028	predicted CorC Co/Mg efflux protein [35]	Cellular processes; Toxin production and resistance	122	58	-1.08	.0007
TPANIC_0333	*lolA*	Cell envelope; lipoprotein transport*	863	406	-1.08	4.14E-12
TPANIC_0833	hypothetical protein	Hypothetical protein	1,109	522	-1.08	2.11E-25
TPANIC_0749	hypothetical protein	Hypothetical protein	26	12	-1.08	.02
TPANIC_RS05255	30S ribosomal protein S20	Ribosomal proteins; Synthesis and modification	102	48	-1.06	4.28E-06
TPANIC_RS05390	hypothetical protein	Hypothetical protein	42	18	-1.06	.007
TPANIC_0404	MBL fold metallo-hydrolase	Hydrolase activity*	147	73	-1.06	1.41E-08
TPANIC_0951	*rpmH*	Ribosomal proteins; Synthesis and modification	28	14	-1.06	.008
TPANIC_0226	hypothetical protein	Hypothetical protein	150	70	-1.05	5.68E-07
TPANIC_0339	*rluA2*	RNA binding*	100	45	-1.05	.002
TPANIC_0786	*lptB*	Transport and binding proteins; Unknown substrate	462	218	-1.03	2.58E-12
TPANIC_0301	ABC transporter permease	Transmembrane transport*	157	72	-1.01	.001
TPANIC_0336	*comE*	Cellular processes; Transformation	278	132	-1.01	6.43E-06
TPANIC_0408	ATPase	ATPase activity*	1,771	3,584	1.00	6.50E-15
TPANIC_0985	*aspS*	Translation; Aminoacyl tRNA synthetases	2,631	5,289	1.01	7.75E-24
TPANIC_0071	*clpB*	Translation; Degradation of proteins, peptides, glycopeptides	2,687	5,436	1.01	1.42E-23
TPANIC_0377	*FliL1*	Cell envelope; Surface structures*	347	697	1.02	5.13E-13
TPANIC_0317	*tprG*	Other categories; Unknown	975	1,973	1.03	8.70E-19
TPANIC_0136	hypothetical protein	Cellular component; Plasma membrane*	2,951	6,103	1.03	9.40E-12
TPANIC_0536	*seccG*	Protein transmembrane transport*	215	441	1.04	5.16E-09
TPANIC_0206	*rpsE*	Translation; Ribosomal proteins: Synthesis and modification	112	227	1.05	4.72E-07
TPANIC_0746	*ppdK*	Energy metabolism; Glycolysis/gluconeogenesis	12,502	25,682	1.05	1.31E-11
TPANIC_0189	*rplC*	Translation; Ribosomal proteins: Synthesis and modification	672	1,372	1.09	6.66E-13
TPANIC_0001	*dnaA*	DNA metabolism; DNA replication, recombination and repair	297	628	1.09	2.81E-13
TPANIC_0870	*flaB3*	Cell envelope; Surface structures	7,160	15,287	1.09	4.15E-13
TPANIC_0379	*secA*	Cellular processes; Protein and peptide secretion	3,552	7,397	1.10	7.75E-20
TPANIC_1029	*dbpA*	Translation; rRNA processing*	2,436	5,649	1.10	3.21E-07
TPANIC_0965	*macA*	Cell envelope; Other	1,074	2,333	1.11	8.26E-17
TPANIC_0195	*rpsC*	Translation; Ribosomal proteins: Synthesis and modification	166	366	1.12	7.05E-14
TPANIC_0346	DUF2715 domain-containing protein	Other categories; Unknown*	415	891	1.12	1.84E-08
TPANIC_0017	hypothetical protein	Other categories; Unknown	884	1,948	1.16	3.54E-13
TPANIC_0319	*tmpC*	Cell envelope; Lipoproteins	682	1,518	1.18	1.81E-14
TPANIC_0188	*rpsJ*	Translation; Ribosomal proteins: Synthesis and modification	112	275	1.18	1.23E-08
TPANIC_0474	YebC/PmpR family DNA-binding transcriptional regulator	DNA metabolism; transcription*	837	1,900	1.19	9.00E-11
TPANIC_0099	*pyrH*	Purines, pyrimidines, nucleosides, nucleotides; Nucleotide and nucleoside interconversion	745	1,798	1.20	2.44E-08
TPANIC_0919	*trxA*	Energy metabolism; Electron transport	1,454	3,417	1.22	9.54E-37
TPANIC_0887	*rpsO*	Translation; Ribosomal proteins: Synthesis and modification	269	681	1.22	.0001
TPANIC_RS05205	hypothetical protein	Hypothetical protein	229	531	1.22	1.17E-14
TPANIC_0193	*rpsS*	Ribosomal proteins; Synthesis and modification	28	59	1.27	.004
TPANIC_0214	hypothetical protein	Hypothetical protein	310	830	1.28	5.94E-05
TPANIC_0461	XRE family transcriptional regulator	DNA binding*	113	340	1.29	.02
TPANIC_RS01765	helix-turn-helix transcriptional regulator	Hypothetical protein	1,037	2,541	1.31	2.91E-39
TPANIC_0991	rubredoxin	Energy metabolism; Electron transport	176	435	1.31	4.34E-11
TPANIC_1032	NusG domain II-containing protein	Hypothetical protein*	1,206	3,387	1.35	7.24E-11
TPANIC_0197	*rpmC*	Ribosomal proteins; Synthesis and modification	26	67	1.38	.0006
TPANIC_0971	*tpd*	Cell envelope; Other	1,024	2,784	1.38	9.24E-12
TPANIC_0744	*prp*	Translation; Ribosomal protein synthesis and modification*	85	214	1.39	5.65E-08
TPANIC_0191	*rplW*	Ribosomal proteins; Synthesis and modification	115	297	1.40	1.99E-06
TPANIC_0939	*nifJ*	Energy metabolism; electron transport	7,087	18,851	1.42	7.12E-33
TPANIC_0202	*rpsN*	Ribosomal proteins; Synthesis and modification	14	45	1.43	.0004
TPANIC_0203	*rpsH*	Ribosomal proteins; Synthesis and modification	131	360	1.43	8.02E-07
TPANIC_0126	hypothetical protein	Cellular component; Cell membrane*	339	948	1.47	2.28E-33
TPANIC_0574	*tp47*	Cell envelope; Biosynthesis of murein sacculus and peptidoglycan	1,979	5,595	1.49	2.46E-15
TPANIC_0941	hypothetical protein	Hypothetical protein	283	846	1.49	4.22E-19
TPANIC_0505	*hxk*	Energy metabolism; Glycolysis / gluconeogenesis	2,741	8,436	1.62	.0002
TPANIC_0869	hypothetical protein	Hypothetical protein	69	232	1.64	6.10E-12
TPANIC_RS01040	*rpmJ*	Translation; Ribosomal proteins: Synthesis and modification	7	25	1.69	.001
TPANIC_0163	*troA*	Transport and binding proteins; Cations	388	1,210	1.70	1.04E-12
TPANIC_0856	UPF0164 family protein	Other categories; Unknown*	1,266	4,722	1.89	5.13E-27

**Table 5:** qRT-PCR validation of *T*. *pallidum* genes with significantly different expression levels *in vitro* and in rabbits. Log_2_ fold difference values are based on the ratio of rabbit to *in vitro*, with negative values indicating that gene expression is lower in rabbits than *in vitro*.

*T*. *pallidum* ORF number	Gene ID	qRT-PCR log_2_ fold difference	RNAseq log_2_ fold difference
TPANIC_1010	*ndk*	-4.63	-1.36
TPANIC_0162	*ruvB*	-3.14	-1.18
TPANIC_0140	*ntpJ*	-2.98	-1.42
TPANIC_0328	*mutS*	-2.51	-1.18
TPANIC_0340	*folC*	-1.50	-1.53
TPANIC_0163	*troA*	2.66	1.70
TPANIC_0505	*hxk*	3.20	1.62
TPANIC_0919	*trxA*	3.81	1.22
TPANIC_0939	*nif*	3.95	1.42
TPANIC_0574	*tp47*	4.01	1.49

### Pathway analysis

To identify potential enrichment of differentially expressed genes in specific biological pathways, protein-coding genes were first annotated with Gene Ontology (GO) terms by homology. Overall 64% (637/1003) of protein-coding genes were successfully annotated with one or more GO terms. Gene set enrichment analyses were then performed using TopGO, ClusterProfiler, and GoSeq. All three analyses identified GO terms representing ribosomal proteins as significantly upregulated in rabbits in comparison to *in vitro* cultures with adjusted p-values of < 0.001. Among GO terms with ten or more members, TopGO also identified ATP metabolic process proteins (p-adj < 0.05) as weakly upregulated in rabbits, whereas DNA repair proteins (p-adj < 0.05), transmembrane transporter activity proteins (p-adj < 0.01), and membrane proteins (p-adj < 0.01) were weakly downregulated in rabbits. Likewise, ClusterProfiler also identified DNA repair proteins (p-adj < 0.05) as downregulated in rabbit cultures. GoSeq did not identify any additional enriched terms. Enrichment was also assessed for a collection of previously identified putative virulence genes [12]; however no significant difference was identified between the rabbit and *in vitro* culture conditions.

### Transport of nutrients

*T*. *pallidum* acquires many protein, nucleotide, and lipid precursors from the environment, and must also maintain appropriate intracellular concentrations of electrolytes and other solutes through transport proteins. However, only a few transporters exhibited differential transcript levels in the rabbit infection and *in vitro* culture models. TPANIC_0163, encoding the ABC transporter periplasmic binding protein TroA that binds iron, zinc, and manganese ions, had one of the highest differential transcription values (log_2_ 1.70, p<1.07 x 10^−12^) between rabbit infection and *in vitro* culture [31–34]. In contrast, transcripts for the magnesium/cobalt efflux proteins TPANIC_0027 and TPANIC_0028 were significantly higher in the *in vitro* samples compared to the rabbit (log_2_−1.17 and -1.08, respectively) [35]. Four additional transport-related genes had significantly higher transcription in the *in vitro* environment: TPANIC_0140 (K^+^ transport protein NtpJ), TPANIC_0840 (major facilitator subfamily [MFS] transporter protein), TPANIC_0786 (ABC transporter ATP binding protein), and TPANIC_0301 (ABC transporter permease).

### Differences in gene transcripts related to metabolism in *T*. *pallidum* cultured in rabbits versus *in vitro*

The transcript levels of *ppdK*, a pyruvate phosphate dikinase, were higher in rabbits than *in vitro*, suggesting that pyruvate metabolism is elevated. The pyruvate-flavodoxin oxidoreductase NifJ (TPANIC_0939), which is thought to be involved in maintenance of a proton gradient across the cytoplasmic membrane, transcript is also elevated during rabbit infection [2]. Related to the redox environment and antioxidant defense, *trxA* (thioredoxin) transcript levels were increased in rabbits compared to *in vitro* culture. The alkyl hydroperoxidase AhpC, also involved in antioxidant defense, has one of the highest transcript levels in both the *in vivo* and *in vitro* environments (Table 3) [36]. Transcripts elevated in *T*. *pallidum* cultured *in vitro* include a gene involved in folic acid biosynthesis (*folC*).

### Varied ribosomal protein gene transcript levels in *T*. *pallidum* cultured in rabbits versus *in vitro*

**Fig 6:**
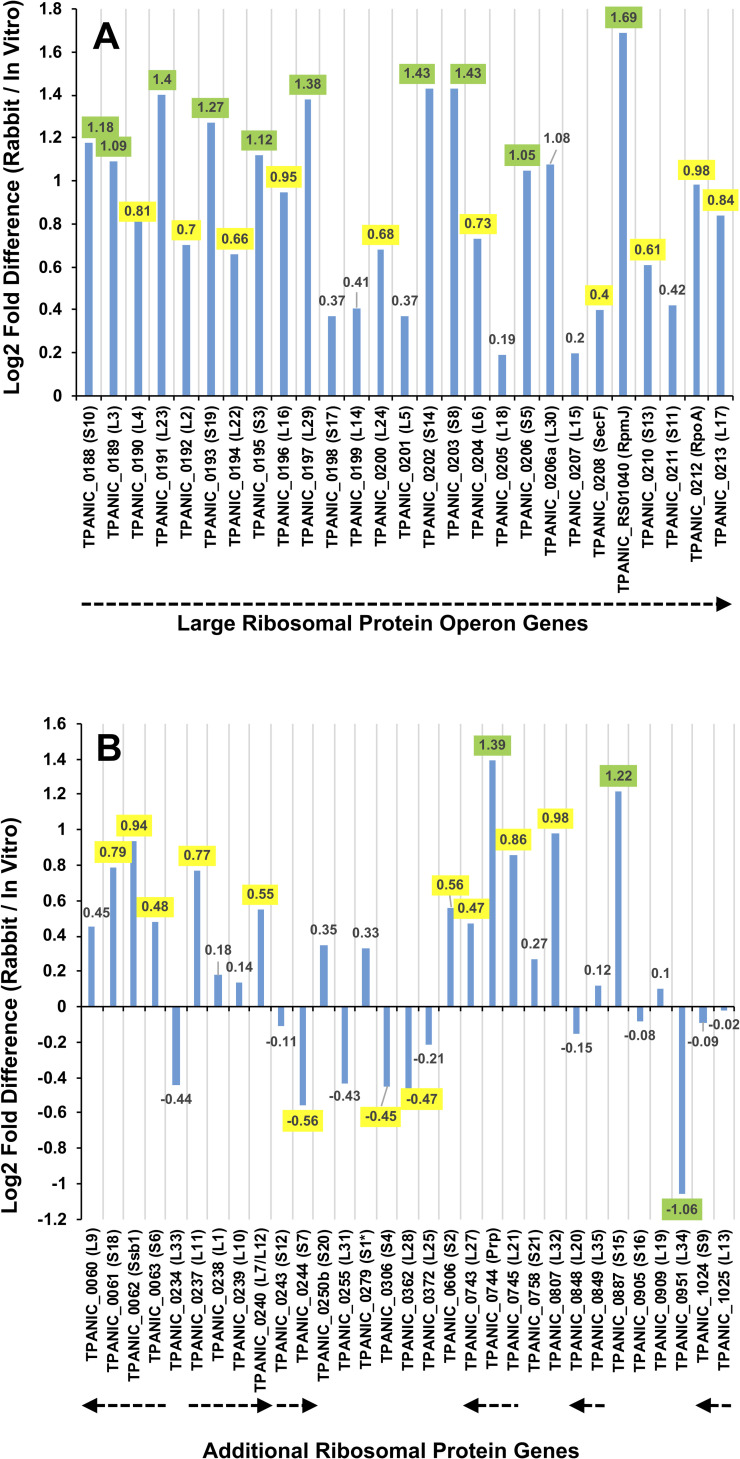
Comparison of differentially expressed ribosomal protein genes between *T*. *pallidum* grown in rabbits and *in vitro*. Log_2_-fold difference and adjusted p-values calculated by DESeq2. Values in green indicate significantly differentially expressed genes with |log_2_-fold difference| of ≥ 1 and p-adjusted of ≤0.05. Values in yellow indicate genes with a significant p-adjusted value of ≤0.05, but without a significant log_2_-fold difference. A) Comparison of genes in the large ribosomal protein operon. B) Comparison of additional ribosomal protein genes.

A total of 14 of 56 ribosomal protein genes had significant differential expression between the long-term *in vitro* cultures and *T*. *pallidum* grown in rabbits, using the criteria of log_2_-fold difference ≥ |1| in transcript levels with a p value ≤0.05 (Table 4). Most of these (11 of 14) had higher transcript levels during rabbit infection as compared to *in vitro* culture. To provide a more comprehensive view, the relative transcript levels for all of the ribosomal protein genes in the large ribosomal protein operon (TPANIC_0188 through TPANIC_0213) and in additional loci were examined (Fig 6). Within the large operon, all 27 genes (including two that do not encode ribosomal proteins) were expressed at a higher level in infected rabbits than in the *in vitro* cultures, with 10 (highlighted in green) of these fulfilling both the 2-fold increase and p≤0.05 significance criteria (Fig 6A). Ten additional genes had p-values less than 0.05 but less than a 2-fold increase (highlighted in yellow). Ribosomal protein genes at other loci (including potential operons of 2–4 genes) had more varied results (Fig 6B). Two additional genes (*ssb1* and *prp*) ‘embedded’ in potential ribosomal protein operons were also included in the results. Only 3 of 31 genes in Fig 6B had ≥1 log_2_-fold differences and p≤0.05, with an additional 11 with smaller differences but p≤0.05. Of the 11 ribosomal protein genes with ≥1 log_2_-fold differences and p≤0.05, 7 encode proteins associated with the 30S ribosomal subunit.

### Membrane and flagellar protein gene transcript levels

Higher transcript levels of putative OmpA-OmpF porin family proteins TPANIC_RS05190 and TPANIC_RS00645 were present *in vitro* than in rabbits. Likewise, transcripts encoding multiple predicted proteins involved in lipoprotein (CoaD and Ddl) and peptidoglycan biosynthesis (MltG), and trans-membrane lipoprotein transport (LolA) were elevated in the *in vitro* samples in comparison to in rabbits. In contrast, levels of transcripts encoding the lactoferrin binding periplasmic lipoprotein Tp34 (TpD) [37], the carboxypeptidase lipoprotein Tp47 [38], and the purine nucleoside-binding lipoprotein [39] PnrA/TmpC were higher in rabbits (Table 4).

Only a few of the genes involved in motility appeared to be differentially expressed in rabbits and *in vitro*. Flagellar assembly is accomplished with 26 known proteins, including three flagellar filament core proteins (FlaB1, FlaB2, and FlaB3), a flagellar filament sheath protein (FlaA1), motor proteins (MotA and MotB), and multiple motor switch proteins (FliG1, FliG2, FliM, and FliN) [40]. In general, there were no consistent differences between flagellar gene transcript profiles in the *in vivo* and *in vitro* environments (S2 Table). Exceptions include the genes encoding FlaB3, FliL1, and FliG1, which exhibited significantly higher transcript levels during infection of rabbits as compared to *in vitro* culture (Table 4).

### Expression of genes involved in DNA replication, transcription and mismatch repair in *T*. *pallidum* cultured in rabbits versus *in vitro*

Transcripts for the chromosome replication initiation protein *dnaA* were more highly expressed during rabbit infection than during *in vitro* culture (Table 4). In contrast, transcripts for the mismatch repair protein *mutS*, the DNA repair and recombination restart protein *recO*, and *ruvB*, a Holliday junction DNA helicase that is also involved in DNA repair, were higher *in vitro* than in rabbits, suggesting that DNA repair processes may be upregulated *in vitro*.

### Regulatory protein gene transcript levels

TPANIC_0474 is highly homologous (51% identical, 73% similar) to the *Borrelia burgdorferi* YebC/PmpR family DNA binding transcriptional regulator (BB0025) that affects the expression levels of VlsE, a *B*. *burgdorferi* surface lipoprotein involved in immune evasion [41]. In our studies, more TPANIC_0474 transcripts were detected in *T*. *pallidum* during rabbit infection as compared to *in vitro*, potentially indicating a regulatory response to the environment in infected rabbits. Similarly, significantly higher transcript levels during rabbit infection were observed with TP_0461, which is predicted to encode a xenobiotic response element (XRE) family regulatory protein with a helix-turn-helix binding motif. None of the five predicted sigma factor genes of *T*. *pallidum* (TPANIC numbers _0493, _0092, _0111, _0709, and _1012) exhibited significantly different transcript levels in the rabbit infection and *in vitro* culture environments (S2 Table).

## Discussion

### Procedural observations

In this study, we compared the transcriptomes of *T*. *pallidum* grown in rabbit testes versus in long-term *in vitro* culture to determine if expression patterns between the two culture conditions are similar. Comparison of the log_2_-transformed FPKM values for rabbit infection and *in vitro* culture showed that RNA transcript levels for these two culture conditions were highly similar. Subsequent differential expression analysis conducted using DESeq2 also indicated that the two culture conditions result in highly similar RNA transcriptional profiles, but significant differences were observed for 94 genes. A subset of these were verified by qRT-PCR. The overall similarity of the transcription profiles during infection of rabbits and *in vitro* culture leads to two possible conclusions. The first is that *T*. *pallidum* is well adapted to its natural, relatively homeostatic environment in tissue and, unlike *Borrelia* species [42], has evolved toward near constant expression of its gene repertoire with few mechanisms of gene regulation (reviewed in [2]). The second possible conclusion is that the conditions in rabbit testicular tissue and those present in the *in vitro* culture system (which, like tissue, includes mammalian cells, a rich source of nutrients, and exposure to microaerobic oxygen levels) are very similar and thus result in closely related transcript patterns. The observed differences in transcript levels may provide insight into genes that are regulated to some degree. It is important to note that in this study, *T*. *pallidum* were obtained from the inoculated, inflamed testes of infected rabbits. It is possible that, related to the systemic nature of syphilis infections, *T*. *pallidum* obtained from other rabbit tissue (such as skin lesions or blood) may exhibit slightly different transcription patterns. Additionally, exposure to more extreme, stressful conditions during *in vitro* culture (e.g. lack of mammalian cells or changes in temperature, oxygen concentration, or medium composition) may also lead to greater differences in gene expression and hence reveal additional regulatory networks.

All of the 1,063 predicted genes were represented in both the rabbit infection and *in vitro* culture transcriptomes, and the majority of the assigned reads were rRNAs. A significant portion of the sequences in all specimens examined did not map to the *T*. *pallidum* genome (29% to 88%); most, if not all, of these populations likely represent rabbit RNA sequences from the infected New Zealand white rabbits or the Sf1Ep cottontail rabbit epithelial cells present in the *in vitro* cultures. In addition, the majority of mapped *T*. *pallidum* RNA sequences corresponded to rRNAs (84% of the assigned *in vitro* sequences and 91% of the assigned rabbit sequences). This result indicates that the RNA preparation kit used for the transcriptome library was not sufficiently effective in enriching for *T*. *pallidum* mRNA; this method uses selective oligonucleotides based on 50 different prokaryotic species to hybridize with and remove prokaryotic rRNA [43,44] and may not work well with *T*. *pallidum* rRNA species. More efficient removal of rabbit cells from the samples prior to RNA extraction, as well as more efficient *T*. *pallidum* mRNA enrichment procedures would be expected to increase the proportion of *T*. *pallidum* sequences recovered in RNA preparations. Similarly, tRNA expression levels were omitted from analysis because the RNA purification, reverse transcription, and cDNA sequencing procedures utilized in this study were not optimal for tRNA recovery and quantitation [45,46].

Comparison of our RNA-seq results with a prior transcriptome analysis utilizing a hybridization procedure [26] showed a low degree of correlation, although there was a general trend with regard to increasing transcript concentration values. The reasons for the relatively poor concordance are unknown, but may be related to differences in RNA preparation procedures or the inherently lower dynamic range and sensitivity of hybridization methods [26,47,48]. The *T*. *pallidum* protein abundance values previously reported by Osbak et al. [30] did not correlate well with our FPKM values obtained by RNA-seq, similar to the poor correspondence that they observed with the previous RNA abundance data obtained by hybridization [26]. In studies with other organisms, R^2^ values between protein and mRNA levels were typically only ~0.4, indicating that post-transcriptional effects may play a major role in the relative abundance of proteins [49].

The RNA-seq results were validated by qRT-PCR of 10 genes that exhibited differential expression (Table 5), and these results had a Pearson R^2^ value of 0.95. The magnitude of expression differences for the 10 genes examined by qRT-PCR were higher than that determined by RNA-seq, possibly indicating that the RNA-seq results may be underestimating transcript levels, but for each gene tested the pattern of expression between rabbit and *in vitro* culture was the same. Although the number of genes in this analysis is limited, the data indicate that the RNA-seq information is useful in comparing transcript levels during rabbit infection and *in vitro* culture conditions.

### Implications for the use of the *in vitro* culture system as a substitute for the rabbit model

Growth and multiplication of *T*. *pallidum* requires acquisition of nutrients, catabolic and anabolic activities, recycling and modification of components (e.g. lipids and nucleotides), synthesis of macromolecules (nucleic acids, proteins, and peptidoglycans), assembly of structures (such as membranes, ribosomes and flagella), and cell division processes. In addition, *T*. *pallidum* has specialized mechanisms to protect it against the host’s immune system, including antigenic variation, limitation of surface immune targets, and adherence and penetration of tissue [2,50]. All of these activities must be regulated to some extent, although the *T*. *pallidum* genome contains only a few genes encoding predicted regulatory factors. Based on the RNA-seq data, transcripts from the genes generally are present in similar levels during rabbit infection and *in vitro* culture. The relative similarity in transcript levels in the two environments support previous work showing that *T*. *pallidum* metabolism and growth is similar during both experimental rabbit infection and the *in vitro* culture system [22,23]. However, the observed transcriptional differences may indicate important effects of these two environments on the organisms.

### Membrane transport and lipoprotein enrichment *in vitro*

*T*. *pallidum* grown *in vitro* demonstrated a weak enrichment of transcripts with GO terms associated with membrane transport. For example, transcripts for genes encoding proteins with predicted involvement in potassium uptake (*ntpJ*) and magnesium/cobalt efflux (TPANIC_0027, TPANIC_0028) [35] were elevated *in vitro* (Table 4), potentially indicating an increased need for balance of these ions in the *in vitro* environment. Conversely, the gene encoding TroA (the periplasmic binding protein of the Fe/Mg/Zn ABC transporter operon *troABCDR* [31–34]) had significantly higher transcript levels during rabbit infection than in *in vitro* cultures. In studies in the related organism *Treponema denticola* [51,52], TroA and the cognate regulator protein TroR were found to be important in ion transport. Therefore, the *tro* operon may play an important role in *T*. *pallidum* metalloregulation and gene expression during infection. Differential expression of genes involved in membrane transport could be due to differing concentrations of important nutrients between TpCM-2 medium and the rabbit model. Interestingly, the testes of 10 month-old rabbits only have about 12% of the zinc concentration found in serum, possibly explaining why *troA* is expressed at higher levels in rabbit testes than it is *in vitro*, and potentially providing a means of nutritional immunity from syphilis infection in the rabbit host [53]. Therefore, this data showing an enrichment of genes involved in membrane transport will be useful for designing future studies aimed at optimizing *in vitro* growth.

Although pathway analysis did not detect a significant difference (p-adj > 0.05) in the expression of genes with GO terms associated with membrane proteins, transcripts were significantly higher *in vitro* for multiple lipoproteins (Oop protein TPANIC_RS05190, and several other predicted membrane lipoproteins) and enzymes involved in lipoprotein and peptidoglycan synthesis (CoaD, Ddl, MltG). Membrane protein genes with significantly higher transcript levels in the rabbit environment included those encoding the Tp47 peptidoglycan carboxypeptidase, lactoferrin-binding lipoprotein Tp34 (TpD), OmpW protein TPANIC_0126, lipoprotein TmpC, Tpr domain-containing protein TPANIC_0017, and the heterogeneous fibronectin-binding lipoprotein TPANIC_0136. These predicted gene products could be involved in the adaptation to the infected rabbit and the *in vitro* culture environments.

### Insights into the metabolism of *T*. *pallidum*

Pathway analysis indicated an enrichment of transcripts for genes associated with ATP metabolic processes in rabbits. For example, elevated expression of *ppdk* in rabbits suggests that pyruvate and phosphoenolpyruvate (PEP) metabolism are elevated in comparison to *in vitro* culture. PEP is thought to be important for the ability of *T*. *pallidum* to respond to differences in amino acid and glucose levels in the environment [2], so increased transcript levels of *ppdk* may allow organisms grown in rabbits to more efficiently switch between utilizing different sources of carbon. As sodium pyruvate is a component of TpCM-2, these data suggest that manipulation of pyruvate levels may be important for *T*. *pallidum*’s growth and survival. In contrast, folic acid biosynthesis or interconversion may be elevated *in vitro* due to an increase in expression of *folC* in comparison to rabbit infection, perhaps indicating that the addition of more folic acid to TpCM-2 may be beneficial. Enrichment of other GO terms involved in metabolic processes was not detected, perhaps indicating that *T*. *pallidum* does not undergo large swings in metabolism when subjected to different culture conditions, which is not surprising due to its highly reduced genome.

### Differences in RNA levels for DNA repair, transcription regulation, and translation machinery genes

Genes with GO terms associated with DNA repair had slight, but significant elevations in transcript levels *in vitro*. For example, transcripts for the mismatch repair protein *mutS* were higher *in vitro* than in rabbits. In the spirochete *B*. *burgdorferi*, MutS is important for repairing the oxidative DNA damage caused by reactive oxygen species (ROS) produced by the infected host [54]. The elevated levels of *mutS* transcripts potentially suggests that *T*. *pallidum* grown *in vitro* may be subject to greater levels of DNA damaging agents (such as ROS) than organisms grown in rabbits [22,54]. Transcript levels of the Holliday junction DNA helicase *ruvB* was also significantly elevated *in vitro*. This protein is activated by the global SOS response to DNA damage in other bacteria; it is possible that the *in vitro* culture system could be inducing higher levels of DNA damage than occur in *T*. *pallidum* grown in rabbits [55].

The *T*. *pallidum* genome encodes very few recognizable regulators; for example, it does not contain any identifiable two-component regulatory systems [2]. One gene that had significantly higher transcript levels in infected rabbits was the transcriptional regulator *yebC*. Zhang et al. [41] found that mutation of *yebC* in *B*. *burgdorferi* resulted in differences in transcript levels in 32 genes, with the largest decrease occurring in the antigenic variation protein gene *vlsE*. The *yebC* mutant was unable to cause long-term infection in immunocompetent mice, most likely due to a deficiency in immune evasion. The YebC ortholog in *T*. *pallidum* (TPANIC_0474) may also affect gene expression, allowing the spirochete to adapt to changing conditions during syphilitic infection, such as increased immune pressure. In addition, TPANIC_0461 is predicted to encode an Xre family [56–59] regulatory protein homolog, and this gene has elevated transcript levels during rabbit infection as compared to in vitro culture (Table 4). Thus it is possible that TPANIC_0461 is capable of altering expression of other genes and aid in adaptation, even in the relatively homeostatic environment of human tissue.

In terms of macromolecular synthesis, pathway analysis found that GO terms associated with ribosomal genes were significantly higher in rabbits; rRNA species were excluded from this analysis, because rRNA was selectively depleted in these preparations to increase the proportion of mRNA reads. Although transcript levels of some of the ribosomal protein genes were significantly elevated, others were not (Fig 5). It is of interest that ribosomal protein transcripts were not consistently upregulated in one growth condition in comparison to the other. There is increasing evidence that ribosome composition can vary between growth conditions or tissues as an additional level of translational control [60–62]; it is possible that *T*. *pallidum* has retained this mechanism as a way of adapting to varied conditions within host tissue. With regard to DNA replication, the chromosome replication initiation protein *dnaA* had significantly higher transcript levels in rabbits than *in vitro*, but transcript levels for other genes necessary for replication (such as the DNA polymerase I, *polA*) were not significantly different. This result suggests that overall transcript levels for genes involved in DNA replication did not differ between the two culture conditions, as supported by our pathway analysis results.

### Implications for future syphilis research

The availability of long-term *in vitro* culture of *T*. *pallidum* [22–25] has opened up the possibility to study the growth, motility, and antimicrobial susceptibility of *T*. *pallidum* without the need for a rabbit host. The similarity of the transcriptomes generated from different culture environments suggests that *T*. *pallidum* does not globally shift its expression levels based on environmental conditions, which is not unexpected for an obligate pathogen with a reduced genome size. Further analysis of the observed differences in gene expression between these two systems may provide insights into adaptive mechanisms that *T*. *pallidum* has retained during genome reduction. One approach is to examine gene expression under different *in vitro* culture conditions, such as varied temperature, pH, medium composition, ROS concentrations, or axenic culture. A future goal for the field is to develop the ability to systematically mutate *T*. *pallidum* and thereby provide a more definitive view of the genetic basis of its unique biology and pathogenesis.

## Materials and methods

### Ethics statement

Rabbit procedures were reviewed and approved by the Animal Welfare Committee of the University of Texas Health Science Center at Houston.

### Bacteria

*T*. *pallidum* subsp. *pallidum* Nichols was originally obtained from J.N. Miller at the UCLA Geffen School of Medicine and cultured *in vitro* in TpCM-2 medium with Sf1Ep cottontail rabbit epithelial cells as previously described [22].

Two male New Zealand White rabbits were inoculated via intratesticular injection with 2–5 x 10^7^ *T*. *pallidum* per testis. Ten days after infection, rabbits were euthanized and the testes were aseptically removed and rinsed in phosphate buffered saline (PBS). Testes extracts were prepared by finely mincing the testes and stirring in extraction buffer (PBS with 20% heat-inactivated rabbit serum and 1 mM DTT, pre-equilibrated with 95% N_2_:5% CO_2_) for 10 min at room temperature, followed by centrifugation at 1000 x g for 2 x 5 min to remove rabbit tissue. The resulting supernatant containing *T*. *pallidum* was treated with RNAprotect Bacteria reagent (Qiagen) to stabilize the RNA and incubated at room temperature for 5 minutes. Bacteria were pelleted by centrifugation for 10 minutes at 10K x g and immediately used for RNA extraction. Approximately 1.6 x 10^10^ *T*. *pallidum* were isolated from each rabbit.

Two sets of three T75 flasks containing Sf1Ep cells and TpCM-2 medium were inoculated with *T*. *pallidum* grown continuously in long-term culture. Organisms were harvested from the flasks after 7 days of *in vitro* growth by removing the TpCM-2 medium and placing it into a 50 mL conical tube, then washing the flask with 2.5 mL trypsin-EDTA and placing the trypsin-EDTA wash into the conical with the reserved TpCM-2 medium. An additional 2.5 mL of trypsin-EDTA was added to the flask, followed by incubation at 37°C for five min to disassociate attached *T*. *pallidum* from the Sf1Ep cells. After trypsinization, the reserved TpCM-2 medium was added back to the flask, pipetted to resuspend the Sf1Ep-*T*. *pallidum* mixture, and returned to the conical tube. The tube was then centrifuged at 100 x g for 7 min to remove the Sf1Ep cells, and the resulting supernatant containing *T*. *pallidum* was immediately treated with RNAprotect Bacteria reagent (Qiagen) for 5 minutes at room temperature. Bacteria were pelleted by centrifugation for 10 minutes at 10K x g and immediately processed for RNA extraction as described below. Each flask yielded ~1x10^9^ *T*. *pallidum*, and was processed separately to provide biological replicates.

### RNA sequencing and data analysis

*T*. *pallidum* RNA was extracted from the rabbit and *in vitro* samples using a Qiagen RNeasy kit as per manufacturer’s instructions. To remove prokaryotic and eukaryotic DNA, on-column DNase digestion was performed using Qiagen RNase-free DNase set. cDNA libraries were prepared with an Ovation Complete Prokaryotic RNAseq Library Preparation kit and sequenced on an Illumina NovaSeq6000 S4 system (150bp paired end reads) by Psomagen Inc. (Seoul, South Korea).

High throughput RNA sequencing reads were preprocessed using Cutadapt v2.3 with parameters set to remove standard Illumina sequencing adapters and enforce a minimum read length of 18 nt. Bowtie2 v2.3.4.1 was used to align the paired-end reads to NCBI RefSeq NC_021490 for *T*. *pallidum* using default parameters with seed substring lengths set to 18 nt [63]. Samtools was used to convert the resulting SAM files to BAM files and to sort the BAM files [64]. The name-sorted BAM files were used to create count tables using HTSeq with filtering set to 0 [65]. DESeq2 and R were used to perform differential expression analysis and to determine statistical significance [66]. GO term annotation was performed using InterProScan v5.36–75.0 [67]. GSEA was performed using adjusted p-value <0.05 as the cutoff for significance and the background gene set as all genes that received adjusted p-values. Default parameters were used with the following exceptions: TopGO v2.40 [68] was run with the weight01 algorithm, ClusterProfiler v3.16 [69], was run with 10,000 permeations and max gene set size of 100, and GOseq v1.40 [70] was run using Benjamini-Hochberg probability corrections. Gene body coverage was calculated in R using RCoverage [71]. These analyses were performed in part using high-performance computing resources of the Texas Advanced Computing Center (TACC) at The University of Texas at Austin.

### Validation of differential expression with qRT-PCR

The same RNA preparations used for RNA sequencing were used for qRT-PCR validation. Primer sets were designed using the Realtime PCR Tool (Integrated DNA Technologies; https://www.idtdna.com/scitools/Applications/RealTimePCR/), setting the PCR product length between 150 and 200 base pairs (S3 Table). An Invitrogen SuperScript First-Strand cDNA Synthesis Kit was used to synthesize cDNA from the *T*. *pallidum* RNA samples following the manufacturer’s directions. qRT-PCR reactions were assembled using 20 ng cDNA and 10 pmol of each primer with an iQ SYBR Green Supermix (Bio-Rad). Reactions were performed on a Bio-Rad C1000 Touch Thermal Cycler using a program of 95°C for 2 min followed by 39 cycles of 95°C for 5 s and 60°C for 30 s. All eight biological samples were run with three technical replicates, no template controls and no RT controls. The resulting data was analyzed by the relative quantification method, where the average ΔΔC_T_ values from the three technical replicates of the gene of interest were normalized to the values of the three technical replicates of the control gene, TPANIC_0426, for each of the 8 biological replicates [26].

## Supporting information

S1 Table Read pairs generated from the *in vitro* and rabbit samples during RNA sequencing. The total number of read pairs per sample and the number of read pairs that map to the *T*. *pallidum* genome were generated by HTSeq with the minimum alignment quality set to 0.
(PDF)

S2 Table Differential expression analysis of the *in vitro* and rabbit samples. Average normalized RNA-seq counts, log_2_-fold difference values (combined rabbit/*in vitro*), and adjusted p-values (combined rabbit vs. *in vitro*) obtained for *T*. *pallidum* subsp. *pallidum* Nichols during *in vitro* culture and rabbit infection. *T*. *pallidum* ORF numbers, gene IDs, and coordinates are from NCBI RefSeq entry NC_021490. ORF numbers in blue indicate genes with significantly higher transcript levels in infected rabbits, while ORF numbers in orange indicate genes with significantly higher transcript levels *in vitro*. * Functional roles based on Gene Ontology (GO) terms (QuickGO). Unless otherwise indicated, functional categories based on [26].
(XLSX)

S3 Table Primer sets used for qRT-PCR. (PDF)
