# Supplementary material for: Comparison of transcriptional profiles of Treponema pallidum during experimental infection of rabbits and in vitro culture: Highly similar, yet different
Source: PLoS Pathog. 2021 Sep 27;17(9):e1009949. doi: 10.1371/journal.ppat.1009949 (PMC8525777; doi:10.1371/journal.ppat.1009949)
Supplement: S1 Table — The total number of read pairs per sample and the number of read pairs that map to the T. pallidum genome were generated by HTSeq with the minimum alignment quality set to 0. (PDF) [file ppat.1009949.s001.pdf]

Sample set	Total read pairs	Assigned read pairs	Unassigned read pairs	rRNA read pairs	mRNA read pairs	tRNA read pairs
***In vitro* Set 1A**	69,512,164	9,616,921 (13.8%)	59,895,243 (86.2%)	8,510,201 (88.5%)	1,100,763 (11.4%)	5,957 (0.1%)
***In vitro* Set 1B**	46,264,513	5,773,537 (12.5%)	40,490,976 (87.5%)	4,832,236 (83.7%)	936,745 (16.2%)	4,556 (0.1%)
***In vitro* Set 1C**	42,629,508	5,356,627 (12.6%)	37,272,881 (87.4%)	4,299,963 (80.3%)	1,051,210 (19.6%)	5,454 (0.1%)
***In vitro* Set 2A**	50,603,212	5,085,803 (10.1%)	45,517,409 (89.9%)	4,233,313 (83.2%)	847,917 (16.7%)	4,573 (0.1%)
***In vitro* Set 2B**	37,361,899	4,759,202 (12.7%)	32,602,697 (87.3%)	3,988,331 (83.8%)	766,749 (16.1%)	4,122 (0.1%)
***In vitro* Set 2C**	34,569,218	4,397,865 (12.7%)	30,171,353 (87.3%)	3,511,882 (79.9%)	881,570 (20.0%)	4,413 (0.1%)
**Rabbit 1**	52,167,004	41,316,442 (79.2%)	10,850,562 (20.8%)	37,324,919 (90.3%)	3,980,507 (9.6%)	11,016 (0.1%)
**Rabbit 2**	36,371,185	21,935,020 (60.3%)	14,436,165 (39.7%)	20,475,539 (93.3%)	1,454,723 (6.6%)	4,758 (0.1%)
