# Supplementary material for: Comparison of transcriptional profiles of Treponema pallidum during experimental infection of rabbits and in vitro culture: Highly similar, yet different
Source: PLoS Pathog. 2021 Sep 27;17(9):e1009949. doi: 10.1371/journal.ppat.1009949 (PMC8525777; doi:10.1371/journal.ppat.1009949)
Supplement: S3 Table — (PDF) [file ppat.1009949.s003.pdf]

**Table S3.** Primer sets used for qRT-PCR.		
***T. pallidum* ORF number**	**qRT-PCR forward primer (5' – 3')**	**qRT-PCR reverse primer (5' – 3')**
TPANIC_0426 [1]	CACCATGACGCAAACGAAGGGTATCGTG T	CTACTCCATCCCCCGTATAATTCC C
TPANIC_0163	CTGTGGTCTTATTCGGTGAAGG	CTGCTGGTACGCCTGATAAC
TPANIC_0505	CGGATATTGCACACCTAGAGAAG	CGTGTACTGCGTGTGAGAAA
TPANIC_0340	CACAACAACGCATGGAATGG	GGGAGAAGGAGACAGATACTAGG
TPANIC_0574	GCATTTCGTGTGGTATCAACTATG	CATCAGCAACTACGTCCCTATAC
TPANIC_0140	CAGAGCGGGCAGTATCTAATC	CACCGGGAAACATCAGAAGA
TPANIC_0939	GTGTGTCCTCACGCAGTTAT	CCCAAATTCCTTGCCTTTGTAG
TPANIC_0919	GGCAGCGGTGTTGTTATTG	GATCGACTTCTTTCCCGTCTT
TPANIC_0162	GGAGGACTACGAACTGGATTG	GTTGCACCAATGAGGGTAAAC
TPANIC_0328	CACACAGCGCAAGGTAATTG	ACAGACGGTGGCTAAGTATTG
TPANIC_1010	GTTCTTCAACGTAGACTGGTAGG	CAACTCCGCAGTAGCTGTATC

Reference:

1. Šmajs D, McKevitt M, Howell JK, Norris SJ, Cai WW, Palzkill T, et al. Transcriptome of *Treponema pallidum*: gene expression profile during experimental rabbit infection. J Bacteriol. 2005;187(5):1866-74. PubMed PMID: 15716460.
